# A New Matrix for Soft Tissue Management

**DOI:** 10.3390/jcm11154486

**Published:** 2022-08-01

**Authors:** Daniele De Santis, Umberto Luciano, Paola Pancera, Giacomo Castegnaro, Christian Alberti, Federico Gelpi

**Affiliations:** Head and Neck Department, Department of Surgery, Dentistry, Pediatrics and Gynecology, University of Verona, 37124 Verona, Italy; daniele.desantis@univr.it (D.D.S.); umbe1@hotmail.it (U.L.); p.pancera9@gmail.com (P.P.); giacomo.castegnaro@gmail.com (G.C.); studiodentisticoalberti@gmail.com (C.A.)

**Keywords:** gingival recession, collagen matrix, connective tissue graft, free gingival graft

## Abstract

Gingival recession is a mucogingival defect defined as the apical shifting of the gingival margin in relation to the CEJ. The use of connective tissue autografts allows for the obtention of very satisfactory results but is associated with undoubted disadvantages. The aim of the present work is to carry out a systematic review of the literature using a meta-analysis to investigate the clinical efficacy of xenogeneic collagen matrix (XCM) in the treatment of gingival recessions. This revision was carried out strictly following the guidelines published in the Cochrane Handbook. Thus, a meta-analysis was performed to calculate relative risks and standardized mean differences for each of the variables considered. The results of the meta-analysis show that CAF + CTG was statistically better than CAF + XCM in almost all the variables analyzed: complete root coverage (RR 0.46), mean root coverage (SMD −0.89), recession reduction (SMD −0.98), clinical attachment level (SMD −0.63) and gingival thickness (SMD −1.68). Meanwhile, CAF + XCM was slightly better than CAF alone in regard to: mean root coverage (SMD 0.51), recession reduction (SMD 0.47) and gingival thickness (SMD 0.56). It is possible to conclude that CAF + CTG still remains the gold standard in radicular coverage.

## 1. Introduction

Gingival recession (GR) is a mucogingival defect defined as the apical shifting of the gingival margin in relation to its physiological position, located 1–2 mm coronally to the cemento-enamel junction (CEJ) [1,2]. This defect is associated with aesthetic impairment because of the exposure of root surface and/or other conditions such as dentinal hypersensitivity and abrasive or carious lesions at the cervical area of the teeth. The treatment of these defects evolved over the years [3,4] until the introduction of bilaminar techniques [5], which consist of the use of a connective tissue graft (CTG) partially or totally covered by a pedunculated flap [6]. According to recent literature reviews, CTG below a coronally advanced flap (CAF) represents the gold standard for the treatment of gingival recessions, as it yields better results in short- and long-term follow-ups [7]. The use of connective tissue autografts in these periodontal surgery procedures yields very satisfactory results even if it is associated with undoubted disadvantages related to: post-operative morbidity, duration of surgery and limited availability of harvested tissue [8]. To overcome these unfavorable conditions, new biomaterials were developed: barrier membranes, enamel matrix derivates [9] and acellular dermal matrix [10,11]. The latter, derived from human donors, is associated with ethical problems and the risk of transmitting infectious diseases [12]. For these reasons, a new xenogeneic collagen matrix (XCM) of porcine origin, indicated for root coverage and other periodontal plastic surgery, has recently been marketed. Different XCMs are produced by different manufacturers, but those most used in the clinical scenario are characterized by a bi-layered structure of type I and III collagen without cross-linking [13]. The compact layer is thin and less permeable to cellular infiltration while the spongy layer is thicker than the latter, and the presence of large pores in the thicker layer allows a huge degree of cellular migration and proliferation [14]. As an alternative, another commercial product exists, consisting of a single layer with interconnected pores and parallel drilling peaks on the side facing the pristine connective tissue in order to promote neo-angiogenesis. Another variant consists of a porcine collagen matrix with resorbable interconnected pores and volumetric stability, thanks to chemical cross-linking [15]. 

Nowadays, the real effect of XCMs on the treatment of GRs is unclear. The aim of the present work is to carry out a systematic review of literature with meta-analysis to investigate the clinical efficacy of XCM in the treatment of GRs.

## 2. Materials and Methods

**Figure 1:**
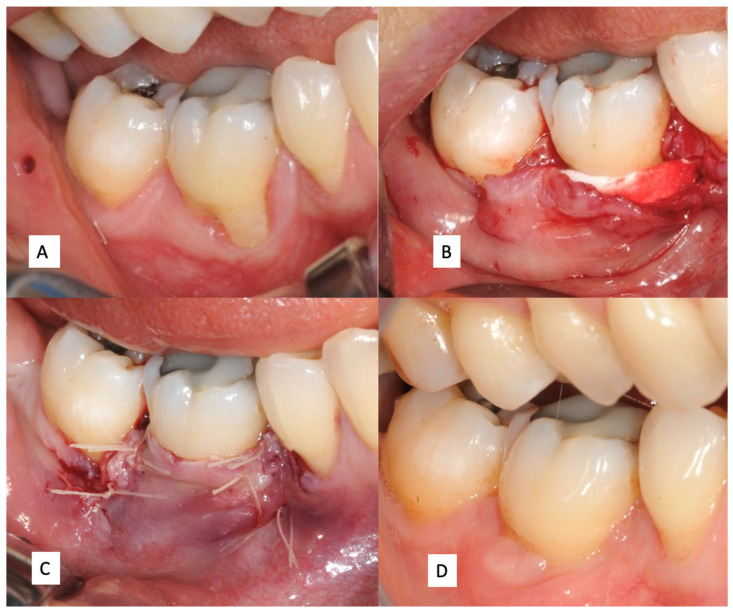
(**A**) Mucogingival defect of the first mandibular molar; (**B**) insertion of the xenogeneic collagen matrix; (**C**) flap suture; (**D**) healing of the surgical site after 6 months.

This study followed the PRISMA statement guidelines [15]. This systematic review was conducted according to the population, intervention, control and outcome (PICO) format. We analyzed clinical trials involving patients with at least one gingival recession classified as Miller I or II (P) who were treated with xenogeneic collagen matrix + CAF (I) or with subepithelial connective tissue graft + CAF or coronally advanced flap alone (C) in order to answer a specific question: the effectiveness of these procedures at least 6 months after surgery (O) (Figure 1).

### 2.1. Focused Question

Is xenogeneic collagen matrix superior than subepithelial connective tissue graft + CAF or coronally advanced flap alone with regard to the treatment of GRs?

### 2.2. Information Sources

Electronic research was performed through the MEDLINE (PubMed) and Cochrane Library databases. In addition, in order to further increase the number of eligible articles and not omit potentially relevant publications, an analysis of the reference list of the main literature reviews and studies performed on the topic of interest was carried out.

### 2.3. Search Strategy

The electronic search was conducted by four independent examiners to minimize reviewer biases, applying the following filters: human studies, date of publication starting 01/01/1998 up to the time of the search (May 2022) and articles published exclusively in English. The search strategy made use of the following terms: “collagen matrix” OR “acellular dermal matrix” OR “dermal matrix allograft” OR “alloderm” OR “keratinized gingiva” OR “keratinized tissue” OR “soft tissue graft” OR “subepithelial connective tissue graft” OR “free gingival graft” OR “mucograft” OR “mucoderm” OR “gingival autograft” OR “attached gingiva” OR “attached mucosa” OR “connective tissue graft” AND “gingival recession”.

### 2.4. Eligibility Criteria

The following inclusion and exclusion criteria were applied in order to carry out study selection.

#### 2.4.1. Inclusion Criteria

- Randomized controlled clinical trials (RCT) and prospective studies with a minimum follow-up of 6 months, which is necessary for the complete healing and maturation of soft tissues subjected to surgery [16];
- Studies with ≥ 5 patients involved;
- Patients with single or multiple GRs classified as class I or II according to Miller 4 or class RT1 according to Cairo et al. [2];
- Studies applying these types of surgery: CAF/tunnel + XCM, CAF/tunnel + CTG, or CAF.

#### 2.4.2. Exclusion Criteria

- In vitro studies, animal studies, retrospective studies, case reports, case series, and systematic reviews;
- Studies with a follow-up < 6 months;
- Studies with < 5 patients involved;
- Patients with single or multiple GRs classified as class III or IV according to Miller [4] or class RT2 or RT3 according to Cairo et al. [2]. We decided to exclude these types of defects as they involve a loss of attachment and bone support at the interproximal level that does not allow for complete and predictable root coverage;
- Surgical interventions other than those previously specified, with biomaterials other than the xenogeneic collagen matrix or with interventions that, although adopting XCM, aimed to compare two different surgical techniques.

### 2.5. Data Items

The variables sought in each study were defined as follows:

- Complete root coverage (CRC), which is a percentage value describing the number of sites, with respect to the total number of sites treated, that obtained a complete radicular covering at a given time of follow-up. The formula to calculate it is the following: $\mathrm{CRC}\;=\left(n.\;\mathrm{of}\;\mathrm{sites}\;\mathrm{with}\;\mathrm{CRC}\right)\;/\;\left(\mathrm{total}\;n.\;\mathrm{of}\;\mathrm{sites}\;\mathrm{treated}\right)\times 100\%$;
- Mean root coverage (MRC), which is a percentage value that describes the rate of reduction of the recession compared to the initial recession;
- Recession reduction (RecRed), which is a millimeter value that describes the difference between the recession measure at a given follow-up and the measure of the initial recession;
- Differential clinical attachment level (ΔCAL), which reflects the gain or loss of CAL at the end of the time of a given follow-up;
- Differential keratinized tissue width (ΔKTW). KTW is the distance from the free gingival margin to muco-gingival junction;
- Differential gingival thickness (ΔGT). GT is a millimeter measurement that indicates the thickness of the attached gingiva.

### 2.6. Study Selection

Titles deriving from the research previously highlighted were reviewed (identification) by two examiners. In the case of disagreement, the two reviewers discussed each case jointly to arrive at a final decision concerning inclusion or exclusion. Articles identified as potentially useful through analysis of the title only were then selected for a more in-depth investigation by reading the abstract. In the examination of the abstract (screening), attention was paid to assessing the compliance of the study with the inclusion criteria. The selected studies were saved as a digital or paper version and submitted to a reading of the full-text (eligibility). In this way, only articles that conformed to the aforementioned criteria were included (included).

### 2.7. Data Extraction

Data extraction was performed by filling in a table with the following data: author, publication year, study design, setting, type of test surgery, type of control surgery, total number of patients, number of test patients, number of control patients, GR type, total number of sites, number of test sites, number of control sites, primary outcomes test, primary outcomes control, secondary outcomes test, secondary outcomes control, patient-reported outcome test (pain, post-operative bleeding or swelling), aesthetics, patient-reported outcome control, follow-up and number of drop-outs.

### 2.8. Quality Assessment

The RCTs included in the meta-analysis were qualitatively assessed using the Cochrane Collaboration tool [17]. The following parameters were adopted for the evaluation of risk of bias: random sequence generation and allocation concealment (selection bias), blinding of participants and personnel (performance bias), blinding of outcome assessment (detection bias), incomplete outcome data (attrition bias), selective reporting (reporting bias) and other possible reasons for bias. 

### 2.9. Meta-Analysis

A meta-analysis was performed, splitting the selected studies into two groups based on the performed surgical technique: (1) CAF + XCM vs. CAF + CTG and (2) CAF + XCM vs. CAF. Since the result of root coverage may follow different temporal trends between the various techniques, only the studies with a 12-month follow-up were included in the meta-analysis. The results of the meta-analysis were expressed as standardized mean difference (SMD) for quantitative variables (MRC, RecRed, ΔCAL, ΔKTW, and ΔGT) and as relative risk (RR) for the qualitative variable (CRC). The magnitude of the SMD was interpreted as mild if SMD = 0.2, mean if SMD = 0.5 and high if SMD = 0.8. [18]. The heterogeneity between studies was assessed with the homonymous test and quantified with the heterogeneity index of Higgins (I^2^), which describes the proportion of heterogeneity of the single studies that cannot be explained by the sampling error and has the advantage of being intrinsically independent from the number of studies. Since the heterogeneity test was significant and/or I^2^ was > 30% [19,20] for all the variables considered, a statistical model with “random” effects was used. Arbitrarily, heterogeneity is considered low if I^2^ is less than 50%, substantial if I^2^ is between 50% and 75% and considerable/high if this parameter exceeds 75%. The pooled estimates and relative confidence intervals were calculated using the DerSimonian and Laird method [21]. The results were graphically represented using a forest plot. The level of statistical significance was set at 5% and confidence intervals (CI) were calculated at 95%. All data was analyzed with the STATA Software (Version 15), StataCorp 4905 Lakeway Drive College Station, Texas 77845 USA.

## 3. Results

### 3.1. Study Selection

**Figure 2:**
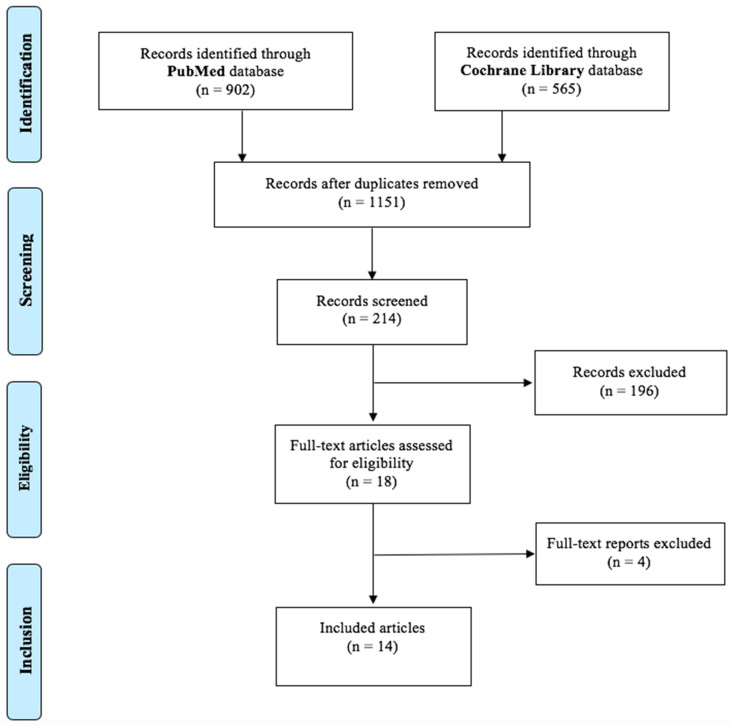
Flow chart of study selection.

The electronic search through the PubMed database identified 902 publications, while the search using the Cochrane Library database identified 565 titles. Following the removal of all duplicates, 1151 articles were identified from 1998 to 2022. Among these, 214 publications were maintained for screening. After the reading of all abstracts, 18 studies positive for eligibility were read entirely (full-text). The reading of full-texts allowed for the exclusion of 4 articles with reason, so the electronic search identified 14 articles, whose data are reported in Table 1 [22,23,24,25,26,27,28,29,30,31,32,33,34,35,36]. A flow chart summarizing the study selection procedure was constructed in accordance with PRISMA guidelines (2009) (Figure 2).

### 3.2. Characteristics of the Included Study

As stated in inclusion criteria, all 14 studies included in this review were randomized controlled clinical trials (RCTs) [22,23,24,25,26,27,28,29,30,31,32,33,34,35,36]. Eight of these articles were performed with a split mouth design, in which each patient was treated with both test and control surgical intervention; in six studies, a parallel groups design was adopted in order to divide patients into two groups treated with the test or control surgery, respectively. All publications compared two types of periodontal surgery for GRs: CAF/tunnel + XCM (test) vs. CAF/tunnel + CTG or CAF alone (control). 

The number of patients included in each study ranged from 8 to 187 and the number of sites treated varied from 8 to 485. The number of patient drop-outs was reported in all articles, most of which were 0. Only four patients from two articles [13,25] dropped out of the study: the number of drop-outs was so low that it did not influence the results. 

In eight studies single GRs were treated, while in the remaining six studies multiple GRs were subjected to surgery. Only few articles reported the reason why patients asked for intervention: in most cases the indications for the intervention were represented by the aesthetic need to mask the mucogingival defect and/or the treatment of dentinal hypersensitivity. For further details see Table 1.

### 3.3. Risk of Bias

**Figure 3:**
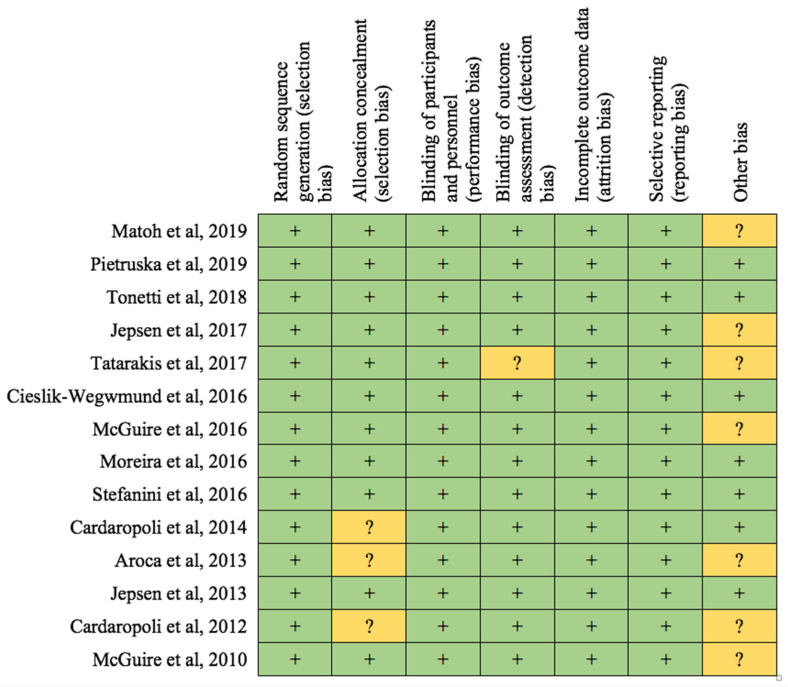
Assessment of risk of bias.

The assessment of the risk of bias within the studies included in this review is summarized in Figure 3. 

Six articles were classified as low risk of bias, while eight publications were classified as unclear risk of bias. In particular, with regard to “random sequence generation”, the risk of bias was adequate in all publications. “Allocation concealment” was generally performed using an opaque envelope containing the type of intervention that the operator had to perform on the patient: this envelope was opened only after the design of the flap on the treatment site. This risk of error was unclear in three articles. The blinding of participants to the type of intervention selected was not possible due to the nature of the intervention itself, which required either the execution of a second surgical site on the palate for collecting the connective graft or the use of the collagen matrix/CAF alone. The blinding of the staff in relation to the type of surgery performed was obviously not possible (the surgeon knows the type of intervention he is carrying out on the patient). Despite this, it is the opinion of the author that performance bias did not compromise the quality of the studies considered, as in oral surgery it is very difficult, if not impossible, to eliminate this bias. For these reasons, it was arbitrarily decided to consider all articles low-risk with regard to performance bias. Only one article [34] was classified as unclear risk of bias relative to “detection bias”. Despite some limited patient drop-outs (4 drop-outs), it is possible to state that there were no incomplete data. Furthermore, no errors were reported in reporting the results. As regards the “other bias” section, it was decided to attribute a risk of unclear bias to the articles in which the collagen matrix was subsidized by the manufacturer.

### 3.4. Results of Individual Studies

The parameter CRC was reported by 13 articles: it ranged from 14.3% to 100% of treated sites. Thirteen studies reported the parameter MRC, which varied in a range from 53.20% ± 32.17% to 99.3% ± 2.54%. The parameter RecRed was present in eight publications and ranged from 1.00% ± 0.69% to 2.95% ± 0.69%. For the values of the other parameters and for further details see Table 2.

### 3.5. Synthesis of Results

In order to reduce the heterogeneity between studies and to improve the quality of the statistics, only the studies with a 12-month follow-up were included in the meta-analysis. There were eight publications included in the meta-analysis [22,23,25,28,29,30,31,32] and six parameters considered (CRC, MRC, RecRed, ΔCAL, ΔKTW and ΔGT). For each parameter, we created a forest plot in order to explore the efficacy of CAF + XCM (test) compared to CAF + CTG or CAF alone (control).

**Figure 4:**
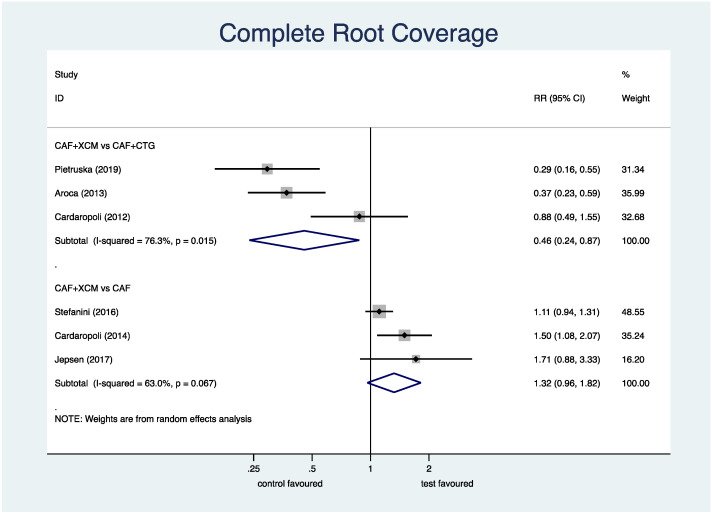
Forest plot relating to complete root coverage.
- Mean root coverage (Figure 5): the statistical analysis shows a statistically significant difference in favor of CAF + CTG compared to CAF + XCM relative to the parameter mean root coverage at 12-months follow-up: SMD (standardized mean difference) −0.89; 95% CI from −1.12 to −0.66; *p* < 0.001. The difference between CAF + XCM and CAF alone is statistically significant in favor of the first surgical procedure: SMD 0.51; 95% CI from 0.002 to 1.01; *p* = 0.049. Notice that SMD is the ratio between the means and the estimated common standard deviation.

- Complete root coverage (Figure 4): the meta-analysis shows a statistically significant difference in favor of CAF + CTG compared to CAF + XCM relative to the parameter of complete root coverage at the 12-month follow-up: RR (relative risk) 0.46; 95% CI (confidence interval) from 0.24 to 0.87; *p* = 0.018. On the contrary, the difference between CAF + XCM and CAF alone is not statistically significant: RR 1.32; 95% CI from 0.96 to 1.82; *p* = 0.085.

**Figure 5:**
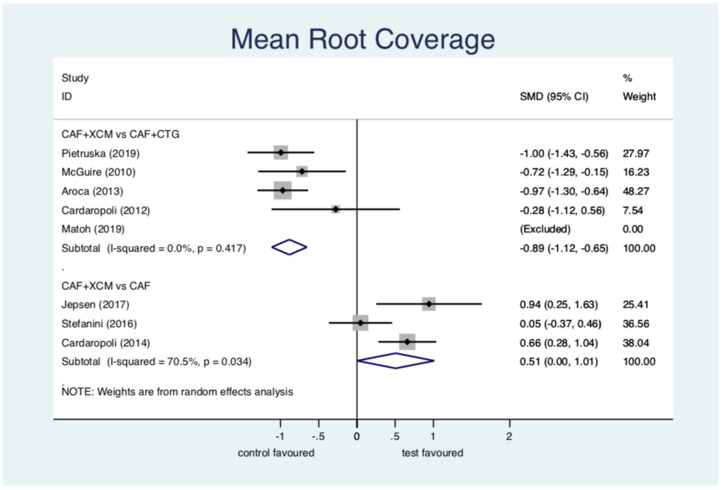
Forest plot relating to mean root coverage.
- Recession reduction (Figure 6): the difference between CAF + XCM and CAF + CTG is statistically significant in favor of the latter procedure relative to the reduction of recession depth 12 months after surgery: SMD −0.98; 95% CI from −1.80 to −0,15; *p* = 0.02. Furthermore, there is a statistically significant difference between CAF + XCM and CAF alone in favor of the first procedure: SMD 0.47; 95% CI from 0.10 to 0.85; *p* = 0.013.

**Figure 6:**
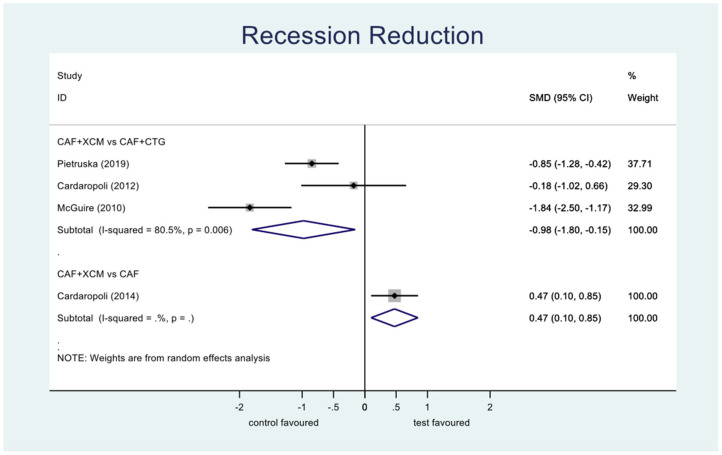
Forest plot relating to recession reduction.
- Differential clinical attachment level (Figure 7): the meta-analysis shows a statistically significant difference in favor of CAF + CTG compared to CAF + XCM relative to the parameter of clinical attachment level at the 12-month follow-up: SMD −0.63; 95% CI from −1.10 to −0.15; *p* = 0.01.

**Figure 7:**
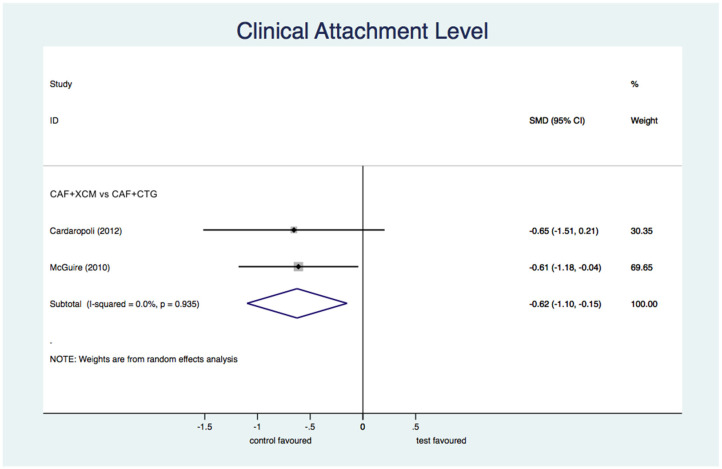
Forest plot relating to the clinical attachment level.
- Differential keratinized tissue width (Figure 8): the difference between CAF + XCM and CAF + CTG is not statistically significant relative to the parameter ΔKTW 12 months after periodontal surgery: SMD −0.68; 95% CI from −2.06 to 0.71; *p* = 0.34. The difference between CAF + XCM and CAF alone is not statistically significant too: SMD 0.27; 95% CI from −0.15 to 0.68; *p* = 0.209.

**Figure 8:**
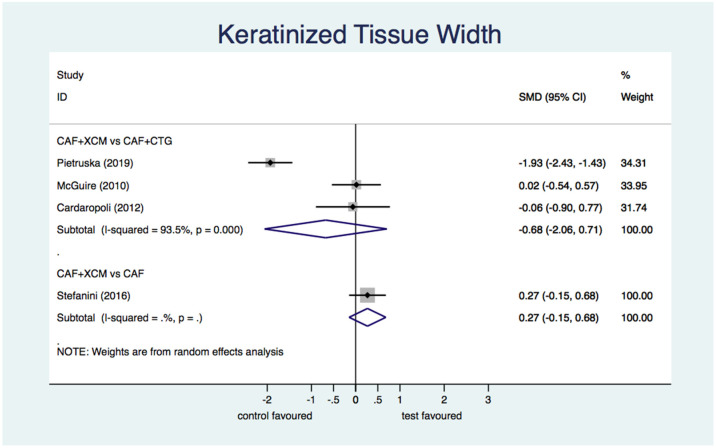
Forest plot relating to keratinized tissue width.
- Differential gingival thickness (Figure 9): the statistical analysis finds a statistically significant difference in favor of CAF + CTG compared to CAF + XCM relative to gingival thickness gain at 12-months follow-up: SMD −1.68; 95% CI from −2.78 to −0.58; *p* = 0.003. The difference between CAF + XCM and CAF alone is statistically significant in favor of the first procedure: SMD 0.56; 95% CI from 0.14 to 0.98; *p* = 0.009.

**Figure 9:**
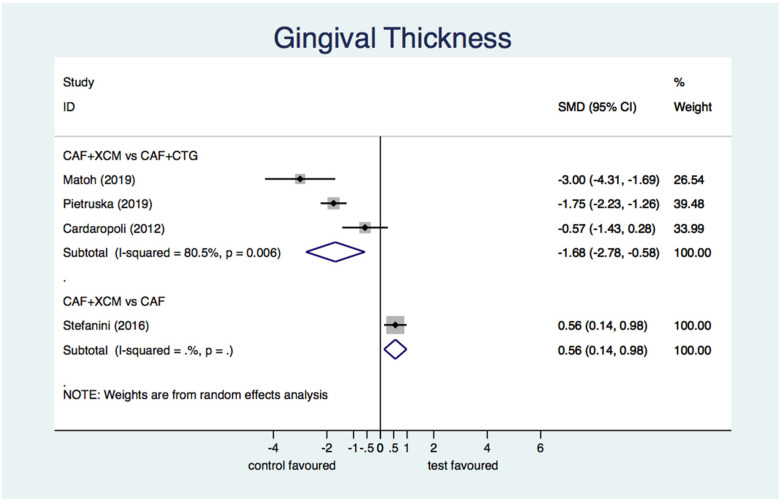
Forest plot relating to gingival thickness.

## 4. Discussion

### 4.1. Quantitative Analysis of Evidence

From a quantitative point of view, the meta-analysis performed in this study stated that CAF + XCM was significantly worse than CAF + CTG with regard to most of the parameters considered (CRC, MRC, RecRed, ΔCAL and ΔGT). In fact, the probability to achieve a complete root coverage with the matrix was halved, and gingival thickness, mean root coverage and recession reduction were decreased by about 1–1.5 standard deviations. Relative to the parameter ΔKTW, the difference between CAF + XCM and CAF + CTG, though in favor of the latter, was not statistically significant.

However, CAF + XCM was slightly superior to CAF alone with respect to gingival thickness, mean root coverage and recession reduction. The increase, although significant, was only about half of the common standard deviation. 

The results of this meta-analysis could be used to realize a rank of the three investigated procedures: the most effective result of CAF + CTG, followed by CAF + XCM and CAF alone, respectively. It is important to emphasize that the meta-analyses were performed considering the results at 12 months because of the lack of wider follow-up data in randomized controlled clinical trials selected from scientific literature. Hence, researchers should make a greater effort to perform delayed clinical evaluations at 24 and 36 months on patients enrolled in randomized clinical trials.

In the scientific literature, there are 16 systematic reviews with meta-analysis on this topic [37,38,39,40,41,42,43,44,45,46,47,48,49,50,51,52]. The two main systematic reviews with meta-analysis are Atieh et al. [37] and Huang et al. [38]. The latter has the disadvantage of not reporting any forest plots or useful graphs with interpretation of the results as well as using data coming from studies with different follow-up periods, while the first publication includes a reduced number of studies. With respect to CAF + XCM vs. CAF + CTG, previous reviews are consistent with the results of this meta-analysis as regards most of parameters considered; the only differences concern MRC (Atieh et al. did not report a statistically significant difference between the two procedures) and ΔKTW (Huang et al. reported a statistically significant difference between the two procedures). With respect to CAF + XCM vs. CAF alone, Huang et al. and Atieh et al. reported results consistent with those of this meta-analysis; the only difference concerns CRC, since Huang et al. indicated that CAF + XCM was statistically better than CAF alone.

### 4.2. Qualitative Analysis of Evidence

Bilaminar techniques are considered the gold standard in gingival recession surgery because of their proven efficacy, which was confirmed by our meta-analysis too. A recent review of literature [9] stated that the procedure of CAF + CTG, analyzed in 28 randomized controlled clinical trials, achieved a mean root coverage of 84.7% and a complete root coverage of 51.8%. Notwithstanding these good results, connective tissue harvesting from a patient’s palate is associated with some disadvantages, such as post-operative pain or discomfort, limited tissue availability and long chair time [8]. Xenogeneic collagen matrix was produced with the aim of overcoming the above-mentioned disadvantages. In fact, in the randomized controlled clinical trial of Tonetti et al. [24], post-operative pain, investigated using a visual analogue scale, was always slightly lower in the group treated with XCM compared to that of the group treated with a connective tissue graft, although these results did not achieve statistical significance; a study published by Aroca et al. [30] investigated post-operative pain through the VAS scale: patients who received collagen matrix complained of a statistically lower pain than patients with connective tissue autografts. Despite the limited scientific evidence, these data suggest that collagen matrix allows for the reduction of post-operative pain in patients treated for root coverage and avoids a second surgical site. Thus, reduction of patient morbidity is one of XCM’s major advantages.

The possibility of avoiding the collection of a connective tissue graft from the palate is also associated with reduction of surgical time, which is a substantial advantage, especially for phobic patients [24,30]. 

Another aspect that could support the use of XCM is the limited availability of autologous CTG, which is a problem in the case of multiple adjacent recessions. 

Finally, as regards patients’ aesthetic satisfaction, in most of selected studies results were slightly higher or comparable with those obtained using a connective tissue graft, although differences between the two types of procedure never reached statistical significance. Therefore, the aesthetic equivalence of XCM and CTG makes both clinicians and patients particularly satisfied about the aesthetics of soft tissue following root coverage with XCM.

### 4.3. Quality and Limitations of Included Studies

The assessment of the reported studies has already been extensively described in the Results Section, to which the authors refer the reader.

## 5. Conclusions

Within the limits of the present work, it is possible to state that the use of xenogeneic collagen matrix under a coronally advanced flap or a tunnel to achieve root covering tends to show a slightly greater efficacy compared to the results of CAF alone. However, the results achieved with CAF + XCM were lower than those with CAF + CTG in relation to all clinical parameters observed. Therefore, it is possible rank these root covering procedures in decreasing order of efficacy:

1. CAF/tunnel + CTG;
2. CAF/tunnel + XCM;
3. CAF.

XCM was worse than CTG in relation to all the clinical parameters analyzed, but it has undoubted advantages that in some situations make it preferable even to autografting. In fact, it avoids the preparation of a donor surgical site for the harvesting of connective tissue (usually from the palate), consequently reducing post-operative morbidity and the duration of surgery. In future investigations we suggest reporting long-term results, even as simply as recalling the patients already treated for the collection of further data. Furthermore, considering the small number of RCTs on the use of XCM in root coverage, further studies are required to evaluate the efficacy of XCM in this type of periodontal surgery with greater significance. 

## Figures and Tables

**Table 1:** Articles included from the study selection.

Authors	Year	Design	Setting	N° Patients	N° Sites	Test Surgery	Control Surgery	Type of Defect	Follow-Up (Months)
Matoh et al. [23]	2019	RCT (split mouth)	University	10	20	CAF + XCM	CAF + CTG	single GRs	12
Pietruska et al. [24]	2019	RCT (split mouth)	University	20	91	tunnel + XCM	tunnel + CTG	multiple GRs	12
Tonetti et al. [25]	2018	RCT (parallel groups)	University	187	485	CAF + XCM	CAF + CTG	multiple GRs	6
Jepsen et al. [26]	2017	RCT (split mouth)	University	18	36	CAF + XCM	CAF	single GRs	36
Tatarakis et al. [35]	2017	RCT (parallel groups)	University	8	8	CAF + XCM	CAF + CTG	single GRs	6
Cieslik-Wegwmund et al. [27]	2016	RCT (parallel groups)	University	28	106	tunnel + XCM	tunnel + CTG	multiple GRs	6
McGuire et al. [28]	2016	RCT (split mouth)	Private practice	17	34	CAF + XCM	CAF + CTG	single GRs	60
Moreira et al. [34]	2016	RCT (parallel groups)	University	40	40	CAF + XCM	CAF	single GRs	6
Stefanini et al. [29]	2016	RCT (split mouth)	University and private practice	45	90	CAF + XCM	CAF	single GRs	12
Cardaropoli et al. [30]	2014	RCT (parallel groups)	Not specified	32	113	CAF + XCM	CAF	multiple GRs	12
Aroca et al. [31]	2013	RCT (split mouth)	University	22	156	tunnel + XCM	tunnel + CTG	multiple GRs	12
Jepsen et al. [36]	2013	RCT (split mouth)	University and private practice	45	90	CAF + XCM	CAF	single GRs	6
Cardaropoli et al. [32]	2012	RCT (parallel groups)	Private practice	18	22	CAF + XCM	CAF + CTG	multiple GRs	12
McGuire et al. [33]	2010	RCT (split mouth)	Private practice	25	50	CAF + XCM	CAF + CTG	single GRs	12

**Table 2:** Parameters used in the articles and their values.

Authors	Year	Surgery Test VS Control	CRC (%)	MRC (%)	RecRed (mm)	ΔCAL (mm)	ΔKTW (mm)	ΔGT (mm)	Follow-Up (Months)
Matoh et al. [23]	2019	CAF + XCM	70	85 ± 24	-	-	-	0.3 ± 0.2	12
		CAF + CTG	100	100	-	-	-	0.9 ± 0.2	
Pietruska et al. [24]	2019	Tunnel + XCM	19.6	53.20 ± 32.17	1.00 ± 0.69	-	0.52 ± 0.65	0.27 ± 0.40	12
		Tunnel + CTG	68.8	83.10 ± 27.63	1.54 ± 0.58	-	2.78 ± 1.53	1.1 ± 0.54	
Tonetti et al. [25]	2018	CAF + XCM	48	-	1.7 ± 1.1	-	−0.1 ± 1.1	-	6
		CAF + CTG	70	-	2.1 ± 1	-	0.5 ± 1.2	-	
Jepsen et al. [26]	2017	CAF + XCM	61.1	91.70 ± 12.05	2.92 ± 0.71	3.17 ± 1.11	1.92 ± 1	0.59 ± 0.39	36
		CAF	38.9	82.77 ± 17.03	2.53 ± 0.72	2.67 ± 1.14	1.03 ± 1.1	0.16 ± 0.40	
Cieslik-Wegwmund et al. [27]	2016	Tunnel + XCM	14.3	91 ± 13	-	-	-	-	6
		Tunnel + CTG	71.4	95 ± 11	-	-	-	-	
McGuire et al. [28]	2016	CAF + XCM	52.9	77.6 ± 29.2	-	-	-	-	60
		CAF + CTG	88.2	95.5 ± 12.8	-	-	-	-	
Moreira et al. [34]	2016	CAF + XCM	40	77 ± 21.2	2.41 ± 0.73	-	-	0.40 ± 0.19	6
		CAF	35	72 ± 14.4	2.25 ± 0.50	-	-	0.14 ± 0.29	
Stefanini et al. [29]	2016	CAF + XCM	93.3	76.28 ± 28.07	2.48 ± 1.46	-	1.06 ± 1.07	0.52 ± 0.46	12
		CAF	84.4	75.05 ± 25.24	2.26 ± 1.17	-	0.64 ± 1.05	0.27 ± 0.43	
Cardaropoli et al. [30]	2014	CAF + XCM	72.4	93.25 ± 10.01	2.28 ± 0.82	-	-	-	12
		CAF	48.1	81.49 ± 23.45	1.85 ± 0.99	-	-	-	
Aroca et al. [31]	2013	Tunnel + XCM	23	71 ± 21	-	-	-	-	12
		Tunnel + CTG	59	90 ± 18	-	-	-	-	
Cardaropoli et al. [32]	2012	CAF + XCM	72	94.32 ± 11.68	2.86 ± 0.39	2.41 ± 0.83	1.23 ± 0.61	1 ± 0.32	12
		CAF + CTG	81	96.97 ± 6.74	2.95 ± 0.69	2.95 ± 0.82	1.27 ± 0.65	1.23 ± 0.47	
McGuire et al. [33]	2010	CAF + XCM	-	88.5 ± 21.08	2.17 ± 0.67	2.26 ± 1.21	1.11 ± 0.82	-	12
		CAF + CTG	-	99.3 ± 2.54	3.17 ± 0.38	2.85 ± 0.63	1.09 ± 1.6	-	

## References

[1] Cortellini P., Bissada N.F. (2018). Mucogingival conditions in the natural dentition: Narrative review, case definitions, and diagnostic considerations. J. Periodontol..

[2] Cairo F., Nieri M., Cincinelli S., Mervelt J., Pagliaro U. (2011). The interproximal clinical attachment level to classify gingival recessions and predict root coverage outcomes: An explorative and reliability study. J. Clin. Periodontol..

[3] Bernimoulin J.-P., Luscher B., Muhlemann H.R. (1975). Coronally repositioned periodontal flap. Clinical evaluation after one year. J. Clin. Periodontol..

[4] Miller P.D. (1985). A classification of marginal tissue recession. Int. J. Periodontics Restor. Dent..

[5] Langer B., Langer L. (1985). Subepithelial Connective Tissue Graft Technique for Root Coverage. J. Periodontol..

[6] Zucchelli G. (2014). Chirurgia Estetica Mucogengivale.

[7] Cairo F., Nieri M., Pagliaro U. (2014). Efficacy of periodontal plastic surgery procedures in the treatment of localized facial gingival recessions. A systematic review. J. Clin. Periodontol..

[8] Griffin T.J., Cheung W.S., Zavras A.I., Damoulis P.D. (2006). Postoperative complications following gingival augmentation procedures. J. Periodontol..

[9] Cairo F. (2017). Periodontal plastic surgery of gingival recessions at single and multiple teeth. Periodontology 2000.

[10] Wei P.-C., Laurell L., Geivelis M., Lingen M.W., Maddalozzo D. (2000). Acellular Dermal Matrix Allografts to Achieve Increased Attached Gingiva. Part 1. A Clinical Study. J. Periodontol..

[11] Wei P.-C., Laurell L., Lingen M.W., Geivelis M. (2002). Acellular Dermal Matrix Allografts to Achieve Increased Attached Gingiva. Part 2. A Histological Comparative Study. J. Periodontol..

[12] Sanz M., Lorenzo R., Aranda J.J., Martin C., Orsini M. (2009). Clinical evaluation of a new collagen matrix (Mucografts prototype) to enhance the width of keratinized tissue in patients with fixed prosthetic restorations: A randomized prospective clinical trial. J. Clin. Periodontol..

[13] Costa D.R., Nicolau R.A., Raniero L.J., Oliveira M.A. (2015). FTIR and SEM analysis applied in tissue engineering for root recovering surgery. J. Biomed. Mater. Res. Part B Appl. Biomater..

[14] Ghanaati S., Schlee M., Webber M.J., Willershausen I., Barbeck M., Balic E., Görlach C., I Stupp S., A Sader R., Kirkpatrick C.J. (2011). Evaluation of the tissue reaction to a new bilayered collagen matrix in vivo and its translation to the clinic. Biomed. Mater..

[15] Thoma D.S., Nänni N., Benic G.I., Weber F.E., Hämmerle C.H.F., Jung R.E. (2015). Effect of platelet-derived growth factor-BB on tissue integration of cross-linked and non-cross-linked collagen matrices in a rat ectopic model. Clin. Oral. Implants Res..

[16] Moher D., Liberati A., Tetzlaff J., Altman D.G. (2010). Preferred Reporting Items for Systematic Reviews and Meta-Analyses: The PRISMA Statement. Int. J. Surg..

[17] Wilderman M.N., Wentz F.M. (1965). Repair of a Dentogingival Defect with a Pedicle Flap. J. Periodontol..

[18] Higgins J.P.T., Green S. (2011). Cochrane Handbook for Systematic Reviews of Interventions.

[19] Cohen J. (1988). Statistical Power Analysis for the Behavioral Sciences.

[20] Higgins J.P.T., Thompson S.G., Deeks J.J., Altman D.G. (2003). Measuring inconsistency in meta-analyses. BMJ.

[21] Higgins J.P.T., Thompson S.G. (2002). Quantifying heterogeneity in a meta-analysis. Stat. Med..

[22] DerSimonian R., Laird N. (1986). Meta-analysis in clinical trials. Control Clin. Trials.

[23] Matoh U., Petelin M., Gašperšič R. (2019). Split-Mouth Comparison of Coronally Advanced Flap with Connective Tissue Graft or Collagen Matrix for Treatment of Isolated Gingival Recessions. Int. J. Periodontics Restor. Dent..

[24] Pietruska M., Skurska A., Podlewski L., Milewski R., Pietruski J. (2019). Clinical evaluation of Miller class I and II recessions treatment with the use of modified coronally advanced tunnel technique with either collagen matrix or subepithelial connective tissue graft: A randomized clinical study. J. Clin. Periodontol..

[25] Tonetti M., Cortellini P., Pellegrini G., Nieri M., Bonaccini D., Allegri M., Bouchard P., Cairo F., Conforti G., Fourmousis I. (2018). Xenogenic collagen matrix or autologous connective tissue graft as adjunct to coronally advanced flaps for coverage of multiple adjacent gingival recession: Randomized trial assessing non-inferiority in root coverage and superiority in oral health-related quality of life. J. Clin. Periodontol..

[26] Jepsen K., Stefanini M., Sanz M., Zucchelli G., Jepsen S. (2017). Long-Term Stability of Root Coverage by Coronally Advanced Flap Procedures. J. Periodontol..

[27] Cieślik-Wegemund M., Wierucka-Młynarczyk B., Tanasiewicz M., Gilowski L. (2016). Tunnel Technique with Collagen Matrix Compared with Connective Tissue Graft for Treatment of Periodontal Recession: A Randomized Clinical Trial. J. Periodontol..

[28] McGuire M.K., Scheyer E.T. (2016). Long-Term Results Comparing Xenogeneic Collagen Matrix and Autogenous Connective Tissue Grafts with Coronally Advanced Flaps for Treatment of Dehiscence-Type Recession Defects. J. Periodontol..

[29] Stefanini M., Jepsen K., de Sanctis M., Baldini N., Greven B., Heinz B., Wennström J., Cassel B., Vignoletti F., Sanz M. (2016). Patient-reported outcomes and aesthetic evaluation of root coverage procedures: A 12-month follow-up of a randomized controlled clinical trial. J. Clin. Periodontol..

[30] Cardaropoli D., Tamagnone L., Roffredo A., Gaveglio L. (2014). Coronally Advanced Flap with and without a Xenogenic Collagen Matrix in the Treatment of Multiple Recessions: A Randomized Controlled Clinical Study. Restor. Dent..

[31] Aroca S., Molnár B., Windisch P., Gera I., Salvi G.E., Nikolidakis D., Sculean A. (2013). Treatment of multiple adjacent Miller class I and II gingival recessions with a Modified Coronally Advanced Tunnel (MCAT) technique and a collagen matrix or palatal connective tissue graft: A randomized, controlled clinical trial. J. Clin. Periodontol..

[32] Cardaropoli D., Tamagnone L., Roffredo A., Gaveglio L. (2012). Treatment of Gingival Recession Defects Using Coronally Advanced Flap with a Porcine Collagen Matrix Compared to Coronally Advanced Flap with Connective Tissue Graft: A Randomized Controlled Clinical Trial. J. Periodontol..

[33] McGuire M.K., Scheyer E.T. (2010). Xenogeneic Collagen Matrix with Coronally Advanced Flap Compared to Connective Tissue with Coronally Advanced Flap for the Treatment of Dehiscence-Type Recession Defects. J. Periodontol..

[34] Moreira A.R.O., Santamaria M.P., Silvério K.G., Casati M.Z., Junior F.H.N., Sculean A., Sallum E.A. (2016). Coronally advanced flap with or without porcine collagen matrix for root coverage: A randomized clinical trial. Clin. Oral Investig..

[35] Tatarakis N., Gkranias N., Darbar U., Donos N. (2017). Blood flow changes using a 3D xenogeneic collagen matrix or a subepithelial connective tissue graft for root coverage procedures: A pilot study. Clin. Oral Investig..

[36] Jepsen K., Jepsen S., Zucchelli G., Stefanini M., de Sanctis M., Baldini N., Greven B., Heinz B., Wennström J., Cassel B. (2013). Treatment of gingival recession defects with a coronally advanced flap and a xenogeneic collagen matrix: A multicenter randomized clinical trial. J. Clin. Periodontol..

[37] Atieh M.A., Alsabeeha N., Tawse-Smith A., Payne A.G.T. (2016). Xenogeneic collagen matrix for periodontal plastic surgery procedures: A systematic review and meta-analysis. J. Periodontal Res..

[38] Huang J.P., Liu J.M., Wu Y.M., Chen L.L., Ding P.H. (2019). Efficacy of xenogeneic collagen matrix in the treatment of gingival recessions: A systematic review and meta-analysis. Oral. Dis..

[39] Barootchi S., Tavelli L., Zucchelli G., Giannobile W.V., Wang H.L. (2020). Gingival phenotype modification therapies on natural teeth: A network meta-analysis. J. Periodontol..

[40] Chambrone L., Salinas Ortega M.A., Sukekava F., Rotundo R., Kalemaj Z., Buti J., Pini Prato G.P. (2018). Cochrane Database. Syst. Rev..

[41] Tavelli L., Ravidà A., Lin G.-H., Del Amo F.S.-L., Tattan M., Wang H.-L. (2019). Comparison between Subepithelial Connective Tissue Graft and De-epithelialized Gingival Graft: A systematic review and a meta-analysis. J. Int. Acad. Periodontol..

[42] Tavelli L., Barootchi S., Nguyen T.V.N., Tattan M., Ravidà A., Wang H.-L. (2018). Efficacy of tunnel technique in the treatment of localized and multiple gingival recessions: A systematic review and meta-analysis. J. Periodontol..

[43] Xu C., Wang Q., Chen J., Wu Y., Zhao LXu C. (2019). Collagen Matrix for Periodontal Plastic Surgery Procedures: A Meta-analysis Update. Int. J. Periodontics Restor. Dent..

[44] Konflanz W., Orth C.C., Celeste R.K., Muniz F.W.M.G., Haas A.N. (2021). Influence of Donor Site and Harvesting Technique of Connective Tissue Graft on Root Coverage Outcomes of Single Gingival Recessions: Systematic Review and Meta-analyses. J. Int. Acad. Periodontol..

[45] Alsarhan M.A., Al Jasser R., Tarish M.A., AlHuzaimi A.I., Alzoman H. (2019). Xenogeneic collagen matrix versus connective tissue graft for the treatment of multiple gingival recessions: A systematic review and meta-analysis. Clin. Exp. Dent. Res..

[46] Ye P., Wei T., Wang Y., Cai Y.-J. (2020). Autologous Platelet Concentrates as Clinical Substitutes for Connective Tissue Graft in the Treatment of Miller Class I and II Gingival Recessions: An Updated Meta-Analysis. Int. J. Periodontics Restor. Dent..

[47] Moraschini V., de Almeida D.C.F., Sartoretto S., Bailly Guimarães H., Chaves Cavalcante I., Diuana Calasans-Maia M. (2019). Clinical efficacy of xenogeneic collagen matrix in the treatment of gingival recession: A systematic review and meta-analysis. Acta Odontol. Scand..

[48] Bhatia A., Yadav V.S., Tewari N., Kumar A., Sharma R.K. (2021). Efficacy of modified coronally advanced flap in the treatment of multiple adjacent gingival recessions: A systematic review and meta-analysis. Acta Odontol. Scand..

[49] Mancini L., Tarallo F., Quinzi V., Fratini A., Mummolo S., Marchetti E. (2021). Platelet-Rich Fibrin in Single and Multiple Coronally Advanced Flap for Type 1 Recession: An Updated Systematic Review and Meta-Analysis. Medicina.

[50] Elangovan S. (2019). Tunneling Technique in Conjunction with Autogenous Graft or Graft Substitutes Is a Predictable Surgical Approach to Achieve Root Coverage in Isolated or Multiple Gingival Recession Defects. J. Evid. Based Dent. Pract..

[51] Li R., Liu Y., Xu T., Zhao H., Hou J., Wu Y., Zhang D. (2019). The Additional Effect of Autologous Platelet Concentrates to Coronally Advanced Flap in the Treatment of Gingival Recessions: A Systematic Review and Meta-Analysis. BioMed Res. Int..

[52] Panda S., Khijmatgar S., Arbildo-Vega H., Das A.C., Kumar M., Das M., Mancini L., Del Fabbro M. (2022). Stability of biomaterials used in adjunct to coronally advanced flap: A systematic review and network me-ta-analysis. Clin. Exp. Dent. Res..

